# Leishmaniasis in Northern Syria during Civil War

**DOI:** 10.3201/eid2411.172146

**Published:** 2018-11

**Authors:** Khalid Rehman, Julia Walochnik, Johannes Mischlinger, Bodour Alassil, Richard Allan, Michael Ramharter

**Affiliations:** Medical University of Vienna, Vienna, Austria (K. Rehman, J. Walochnik, J. Mischlinger, M. Ramharter);; Bernhard Nocht Institute for Tropical Medicine, Hamburg, Germany (J. Mischlinger, M. Ramharter);; University Medical Center Hamburg-Eppendorf, Hamburg (J. Mischlinger, M. Ramharter);; The MENTOR Initiative, West Sussex, UK (B. Alassil, R. Allan)

**Keywords:** leishmaniasis, conflict, epidemiology, Syria, civil war, Aleppo boil, parasites, Leishmania

## Abstract

Since the onset of the ongoing civil war in Syria, the governmental surveillance system for leishmaniasis has lost access to provinces of northern Syria. The MENTOR Initiative, an international not-for-profit organization, was commissioned to implement an integrated leishmaniasis control program, providing an opportunity to reassess the epidemiology of leishmaniasis in northern Syria. Epidemiologic data and biologic samples for molecular species diagnostics were collected from collaborating local health centers. Incidence peaked in March 2015 at 7,743 estimated monthly cases. High levels of transmission were observed in traditional endemic regions but extended to previously hypoendemic regions, such as Al-Raqqa and Al-Hasakah. Incidence decreased to 3,209 in July 2015. Data indicate that the prewar trend of increasing incidence of cutaneous leishmaniasis accelerated during the beginning of armed conflict but declined after implementation of the comprehensive control program by the MENTOR Initiative. Molecular analysis revealed a spectrum of *Leishmania* species and sporadic cases of visceral leishmaniasis.

Leishmaniasis is caused by different *Leishmania* species. The infection may be cutaneous, mucocutaneous, or visceral, depending on the species involved and the immune status of the patient. The common term Aleppo boil indicates the historic importance of cutaneous leishmaniasis (CL) in Syria. Before the ongoing civil war began, the national leishmaniasis control program in Syria delegated general treatment responsibilities to primary healthcare centers (HCCs) and other health services of the governmental health system. Official World Health Organization (WHO) statistics indicate that ≈14,000 new CL cases occurred per year during 1994–2000. Incidence increased after that, to 27,825 in 2010. In addition to the main burden of CL, sporadic cases of visceral leishmaniasis (VL) (25–55 cases/y) were reported to governmental authorities (*1*).

After the onset of civil war, reports emerged about a dramatic increase in CL cases in Syria and neighboring Jordan (*2**–**4*). These reports were based largely on anecdotal observations of clinical cases in refugees from Syria. However, because of a loss of access of governmental authorities to leishmaniasis-endemic regions within Syria, no reliable epidemiologic data have been recorded and published since the onset of the civil war. In addition, international organizations initially providing treatment structures for CL within Syria operated with conflicting case definitions and reporting systems for leishmaniasis, thus bringing forth unreliable epidemiologic data.

The MENTOR Initiative (http://thementorinitiative.org), an international humanitarian organization, was commissioned to plan and implement a comprehensive leishmaniasis control program in northern Syria. The organization’s principal aim is to relieve tropical diseases in humans, with a focus on vector-borne diseases in the context of complex emergencies. Activities in Syria were launched in September 2013, and a comprehensive control program was set up with both a preventive and a curative component. To provide firm evidence for the magnitude of the leishmaniasis epidemic and to accompany and evaluate the rollout of the leishmaniasis control program, an epidemiologic surveillance system was established. The principal aims of this system were to collect reliable data for the epidemiology of leishmaniasis, to investigate the comparative distribution of *Leishmania* species in clinical samples, and to evaluate diagnostic and therapeutic activities of affected populations in northern Syria.

## Materials and Methods

The leishmaniasis control program was based on current WHO recommendations for integrated vector control management (*5*) aimed at reducing both the vector density and parasite reservoirs. Sand fly populations were targeted by large-scale implementation of indoor residual spraying, provision of long-lasting insecticide-treated nets, long-lasting insecticide-treated curtains, and waste management. The leishmanial parasite reservoir was targeted by providing of curative services for local populations.

### Organizational Setup, Study Area, and Health Infrastructure

Because of the precarious, ever-changing security situation in northern Syria, the MENTOR Initiative opted to not deploy external, international personnel into the conflict zone. A headquarters for coordination of activities was set up in the border region between Syria and Turkey. Implementation of the control program was based on voluntary collaboration of local Syrian HCCs, which had been detached from governmental administration and supply since the onset of the civil war. Healthcare workers in Syria were invited to travel to the mission’s headquarters, where they were offered logistical support and training in the diagnosis and treatment of CL and VL. Pentavalent antimonial therapy was provided to healthcare workers for treating patients at their HCCs.

### Collection of Epidemiologic Data for Northern Syria Provinces

Epidemiologic, clinical, and demographic data were collected from all collaborating HCCs in the target regions. Healthcare workers cleaned lesions with antiseptic solutions and then scraped the margins of ulcerated wounds with sterile lancets. The scraped material was transferred to a glass slide, then fixed with methanol and stained with Giemsa stain. Diagnosis of CL was established by trained healthcare workers at each HCC. Where appropriate laboratory facilities were available, healthcare workers used light microscopy to detect amastigote forms of *Leishmania* spp. (*6**,**7*). Rapid tests (IT-LEISH Individual Rapid Test; Bio-Rad Laboratories, Hercules, CA, USA) were used for diagnosis of VL (*8*).

### Molecular–Epidemiologic Survey of Cutaneous Leishmaniasis at Sentinel Sites

For confirmation of the accuracy of the HCC diagnostic services and to obtain molecular–epidemiologic evidence, skin samples were collected at sentinel sites and transported to headquarters for further analysis. Two representative sentinel sites were selected for this molecular epidemiologic substudy in the provinces of Idlib (Heish HCC) and Aleppo (Batranah HCC).

Heish HCC was located in rural Idlib in a district called Al Ma’ra. During the survey period in 2015, the catchment population of this HCC was 5,933, and the population of internally displaced persons (IDPs) was 1,367. Most of the IDPs had come from Hama and southern Aleppo, because Heish was relatively secure in comparison with those areas. Batranah HCC was located in the Jebel Saman district in southeastern Aleppo. The catchment area of this HCC had a population of ≈3,500, with <500 IDPs. All patients who arrived at these sites during the period of molecular data collection were invited to participate.

Sampling involved first the cleaning of the affected skin with moistened gauze. Then, sterile filter paper was pressed against the moist base and the margins of the ulcer to allow for absorbing wound secretions (*9**,**10*).

### Molecular Analysis of Skin Samples

Biologic samples were shipped to the Medical University of Vienna (Vienna, Austria) for further analysis. We extracted DNA using the QIAmp DNA Mini Kit (QIAGEN, Vienna) according to the manufacturer’s instructions for dried blood spots.

We tested all samples by a universal *Leishmania* PCR protocol using the LITSR/L5.8S primer pair (*11*) and following the protocol of Schönian et al. (*12*). To further discriminate species within the *L. donovani/infantum* complex, we ran 3 more PCR protocols for species differentiation: K26-PCR (*13*), cpbE/F-PCR (*14*,*15*), and HSP70-PCR (*16*) (Technical Appendix).

### Ethics Considerations, Data Collection, and Data Analysis

Because there was no operational, accredited ethics committee for the northern provinces of Syria, we submitted the study protocol to the ethics committee of the Medical University of Vienna. Oral consent was obtained from all patients before study-related procedures. Because of the extraordinary security situation and fear of causing a potential risk for the life of participants if there were a written record of personal information, in the light of activities by the rebel groups, investigators decided not to obtain written informed consent or any identifiable personal information in study-related documentation. The ethics committee approved this approach. 

### Data Analysis 

We performed descriptive data analysis using JMP (https://www.jmp.com*)* and STATA/SE15.0 (https://www.stata.com). We used Microsoft Office (Microsoft, Redmond, WA, USA) to perform data visualization.

## Results 

### The MENTOR Initiative’s Leishmaniasis Control Program in Northern Syria

The MENTOR Initiative’s first target regions were the provinces of Aleppo and Idlib; the group subsequently expanded its activities to Al-Raqqa, Al-Hasakah, and Hama. It was a challenge to reliably estimate the total population residing in the target regions because of the frequent and unpredictable movements of persons after military and political actions and the lack of a stable administrative control over the region.

The MENTOR Initiative supported a network of ≈200 HCCs, of which 161 HCCs provided reliable data over an extended follow-up period. These health facilities included HCCs and mobile clinics in each province (43 and 21 in Aleppo, 40 and 18 in Idlib, 7 and 12 in Hama, 7 and 5 in al-Raqqa, 8 and 0 in Al-Hasakah, respectively). The approximate geographic and population coverage in the target regions is provided in Table 1.

**Table 1:** New cases of cutaneous leishmaniasis diagnosed at collaborating health centers, northern Syria

Province	New cases observed during study period	Health centers	Mobile clinics	% Program coverage of area	New cases estimated*	New cases reported in 2008 (*1,17*)	Incidence ratio
Aleppo†	12,296	43	21	70	13,174	18,603	0.7
Idlib†	21,451	40	18	80	20,110	3,883	5.2
Hama†	10,103	7	12	50	15,155	2,219	6.8
Al-Raqqa‡	5,546	7	5	65	11,376	290	39.2
Al-Hasakah§	439	8	0	45	4,683	290	16.1
Total	49,835	105	56		64,498	25,285	2.6
*Standardized cases for 1 year per province. †16-month observation period. ‡9-month observation period. §2.5-month observation period.							

### Epidemiology of CL in Northern Syria

**Figure 1:**
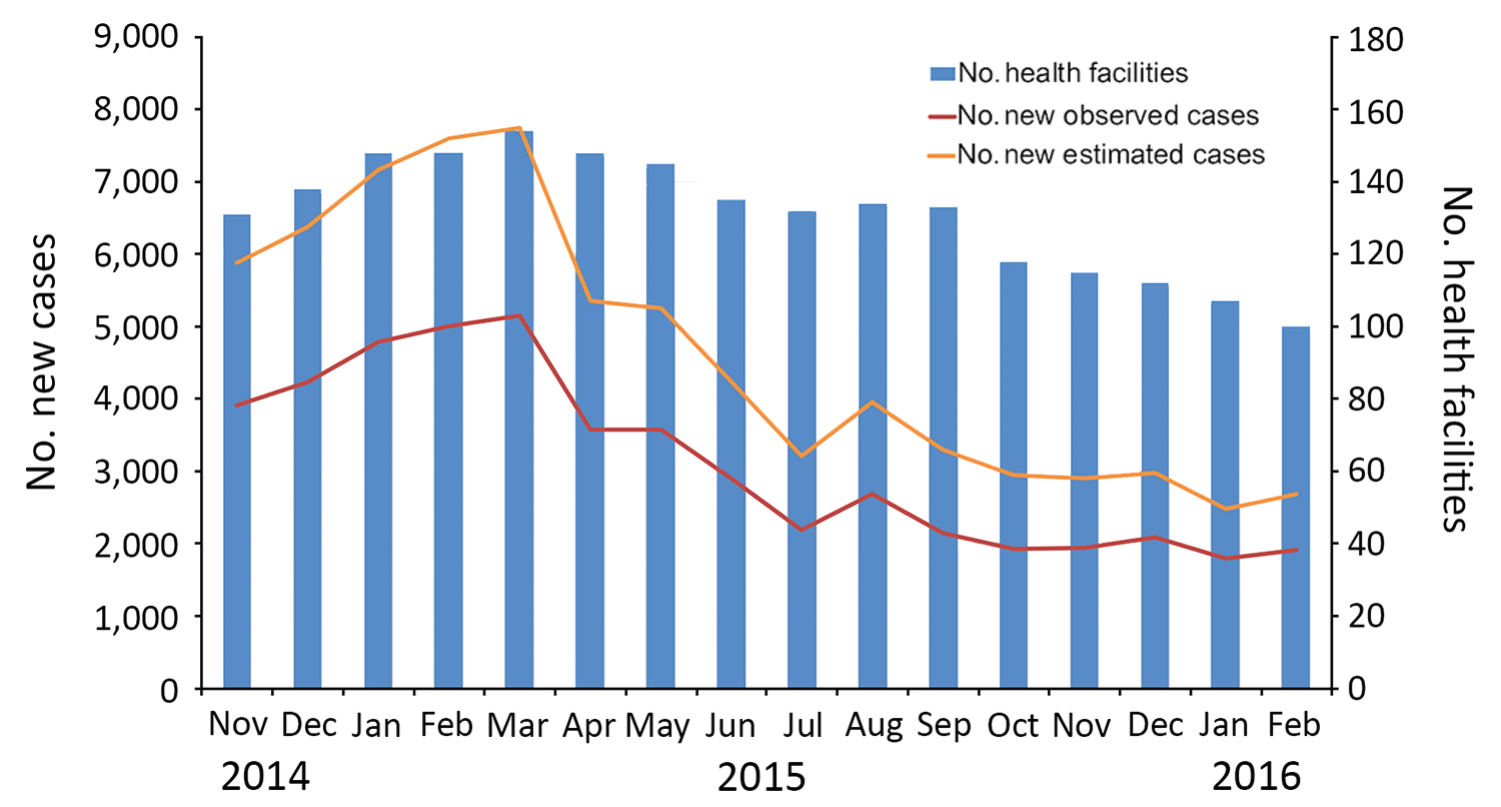
Number of observed and estimated cases of cutaneous leishmaniasis diagnosed in healthcare centers in target regions for leishmaniasis control programs, northern Syria, November 2014–February 2016.

**Figure 2:**
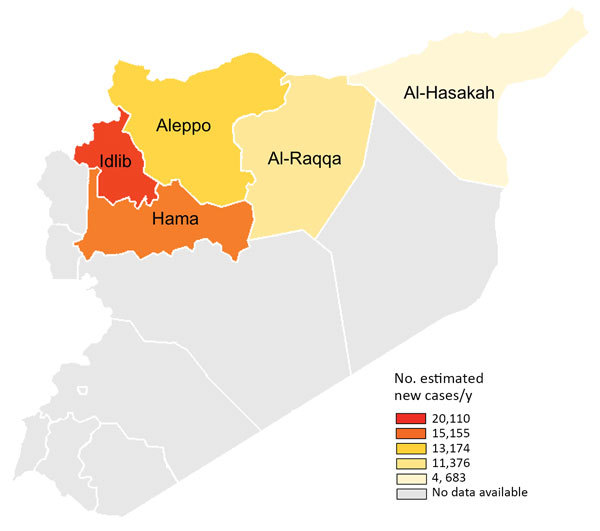
Target region for leishmaniasis control programs in northern Syria (color shading) and the number of estimated new cases of cutaneous leishmaniasis per year by province in this region.

The number of HCCs varied over the study period, mostly because of the changing security situation, which, in turn, led to movement of persons and, in some cases, loss of access to these health facilities. New cases of CL were recorded in HCCs in the provinces of Aleppo, Idlib, Hama, Al-Raqqa, and Al-Hasakah over varying periods, ranging from 2.5 months to 16 months (Table 1; Figures 1, 2). The total number of new cases per year has been estimated based on the observation period and the estimated coverage of HCCs participating in the reporting system of the MENTOR Initiative. Data indicate a yearly incidence of 4,683–20,110 cases/year in these provinces. The total number of new CL patients was estimated at 64,498 cases in 2015 (Table 1).

Data indicate that the estimated incidence of CL was 5,883 cases/month at the onset of the study in the target region. This incidence further increased to ≈7,599 cases/month at its peak in February 2015. After the effective rollout of the leishmaniasis control program, the monthly incidence of CL was reduced to 2,476 cases/month in February 2016 (Figure 1).

Treatment outcome was assessed in 45,302 patients during the study period. Among those, 18% (8,312) were lost to follow-up, and therefore no outcome could be attributed. Among the remaining 36,990 patients, a favorable outcome was recorded by the treating healthcare workers in 35,931 (97.14%, 95% CI 96.96%–97.30%).

### Molecular–Epidemiologic Survey of CL at 2 Sentinel Sites

Of the 249 patients who agreed to participate in this substudy, 104 (42%) were female and 145 (58%) were male, and 139 (56%) had skin lesions suggestive for CL. The median age of participants was 11 years (interquartile range [IQR] 6–17 years), and the median body mass index was 19 (IQR 16–25) (Table 2). In Aleppo, only patients with CL participated in this study. However, the survey had to be prematurely discontinued in Aleppo after the recruitment of 55 participants because of the worsening security situation in the area at the time of the study. 

**Table 2:** Description of participants in the molecular epidemiologic substudy, northern Syria*

Characteristic	Total participants, N = 249

Age, y	
Median (IQR)	11 (6–17)
<10	107 (43)
11–20	86 (34.5)
21–30	22 (8.8)
31–40	11 (4.4)
41–50	7 (2.8)
51–60	7 (2.8)
>61	9 (3.6)
Sex	
M	145 (58.2)
Fe	104 (41.8)
Body-mass index, kg/m^2^	
Median (IQR)	19 (16–25)
Presence of skin lesions, n = 139	
Idlib	84 (60.4)
Aleppo	55 (39.6)
Absence of skin lesions, n = 110	
Idlib	110 (100)
Aleppo	NA
*Values are no. (%) except as indicated. IQR, interquartile range; NA, not available	

Samples were taken from skin ulcers in 139 patients; 99 specimens were successfully shipped to Vienna (40 samples were lost as a result of security problems at the border crossing between Syria and Turkey). We confirmed *Leishmania* spp. infection in 93 (94%) of the 99 samples using PCR. Most *Leishmania* infections were caused by strains of *L. tropica* (n = 73, 78% [95% CI 69%–86%]). We detected *L. major* in 12 samples (13% [95% CI 7%–21%]). Seven infections were caused by *L. infantum* and 1 strain was identified as *L. donovani* (combined proportion of *L. donovani/infantum* complex 9% [95% CI 4%–16%]). The relative proportion of *L. infantum* cases was higher in Idlib province (n = 6, 86% [95% CI 42%–100%]) than in Aleppo province (n = 1, 14% [95% CI 0%–58%]) (Table 3).

**Table 3:** Molecular epidemiology of *Leishmania* spp. in samples collected at sentinel sites, Northern Syria*

Category	*L. tropica*	*L. major*	*L. infantum*	*L. donovani*
Province				
Idlib	50 (83, 71–92)	3 (5, 1–14)	6 (10, 4–21)	1 (2, 0–9)
Aleppo	23 (70, 51–84)	9 (27, 13–46)	1 (3, 0–16)	0 (0, 0–11)
Sex				
M	43 (80, 66–89)	5 (9, 3–20)	5 (9, 3–20)	1 (2, 0–10)
F	30 (77, 61–89)	7 (18, 8–34)	2 (5, 1–17)	0 (0, 0–9)
Age, median (IQR)	13 (7–24)	7.5 (4.5–19)	10 (6–25)	7 (7–7)

*Values are no. (%, 95% CI) except as indicated. IQR, interquartile range.

**Figure 3:**
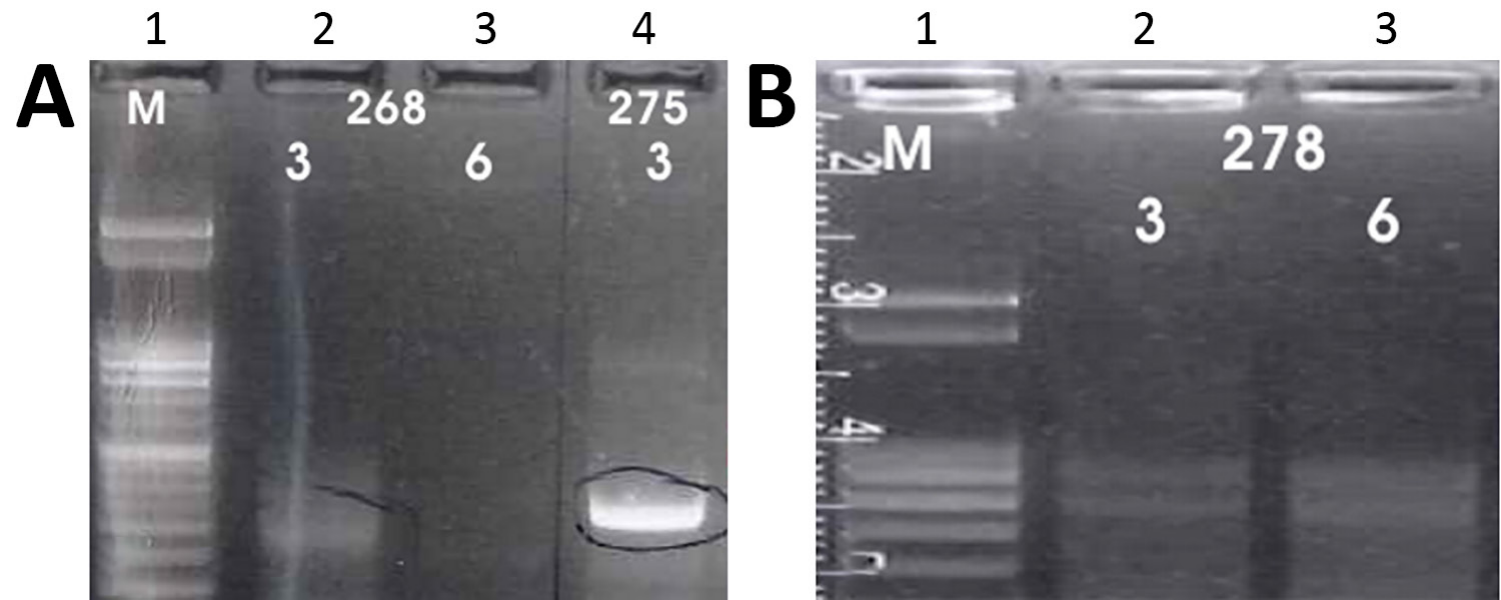
A) Results of K26 PCR assay (*13*) on patient samples identified as belonging to the *Leishmania infantum/donovani* complex in study of leishmaniasis control programs in northern Syria. Lane 1, step marker; lanes 2 and 3, *L. infantum* strain 268 with 3 and 6 μL of DNA, respectively; lane 4, *L. donovani* strain 275 with 3 μL of DNA. B) Results of K26 PCR assay (*13*) on patient samples identified as belonging to the *Leishmania infantum/donovani* complex in study of leishmaniasis control programs in northern Syria. Lane 1, step marker; lanes 2 and 3, *L. infantum* strain 278 with 3 and 6 μL of DNA, respectively.

**Figure 4:**
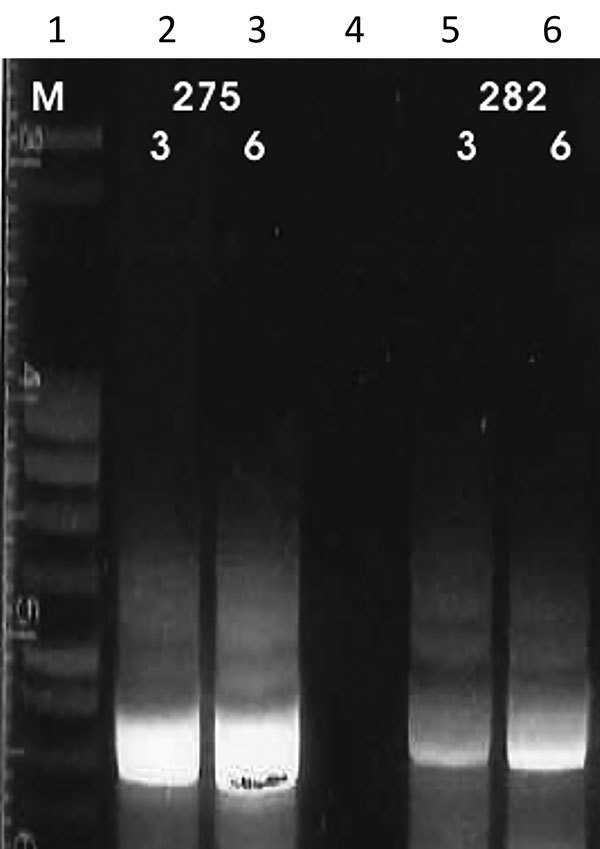
Results of cpb-EF PCR (*14*,*15*) on patient samples identified as belonging to the *Leishmania infantum/donovani* complex in study of leishmaniasis control programs in northern Syria. Lane 1, step marker; lanes 2 and 3, *L. donovani* strain 275 with 3 and 6 μL of DNA, respectively; lane 4, negative control; lanes 5 and 6, *L. infantum* strain 282 with 3 and 6 μL of DNA, respectively.

**Figure 5:**
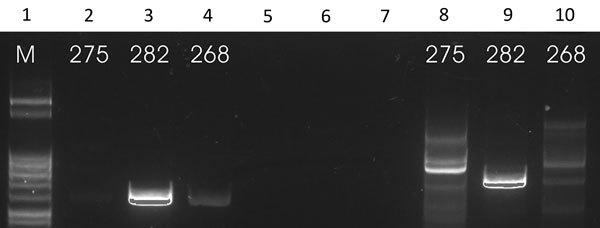
Results of HSP-70 PCR (*16*) on patient samples identified as belonging to the *Leishmania infantum/donovani* complex in study of leishmaniasis control programs in northern Syria. Lane 1, step marker; lanes 2–4, N fragment of *L. donovani* strain 275 and *L. infantum* strains 282 and 268; lanes 5–7, blank; lanes 8–10, T fragment of *L. donovani* strain 275 and *L. infantum* strains 282 and 268.

We performed the K26 PCR successfully in 5 of 8 samples identified as belonging to the *L. infantum/donovani* complex. Retrieved bands were ≈280 bp in 1 case (*L. infantum*, strain 268) and ≈370 bp in 1 case (*L. donovani*, strain 275) (Figure 3, panel A); the other samples produced double bands at ≈800/900 bp (Figure 3, panel B). We also used cpbEF-PCR on the *L. donovani* strain, producing bands of ≈400 bp, and on 1 *L. infantum* strain, producing bands of ≈360 bp (Figure 4). We performed HSP70 PCR with the *L. donovani* strain and 1 *L. infantum* strain (Figure 5). To further confirm and investigate the identity of the *L. donovani* strain, we obtained DNA sequences for the ≈370-bp band of the K26 PCR and the 2 fragments of the HSP70 PCR (together ≈1280 bp). The 368-bp K26 sequence showed highest sequence similarity (358/371 bp) to a *L. donovani* strain isolated from a patient in Israel (GenBank accession no. HQ170543), and the 1,286-bp HSP70 fragment showed 100% similarity (562/562 bp) to the 562-bp fragments available from 4 *L. donovani* strains isolated from CL patients in Turkey close to the Syria border and 1 strain isolated from a CL patient in Cyprus (GenBank accession nos. KU949373–77) and >99% similarity (1,283/1,286 bp) to the *L. donovani* strain pasteur (GenBank accession no. CP022643) and several other *L. donovani* strains. We submitted sequence data obtained in this study to GenBank (accession numbers pending).

### Cases of VL at Collaborating HCCs

During the study period, 11 cases of VL were reported. Seven patients were male, and 7 were children <5 years of age (range 3 months–39 years). Seven patients were reported from Aleppo province and 4 from Idlib. Seven patients had ongoing fever and 1 patient high-grade fever. Anemia was reported for 6 patients and hepatosplenomegaly for 3 patients. Five patients additionally underwent bone marrow aspiration and showed positive PCR results for *L. infantum* infection. All patients with diagnoses of VL were treated systemically with pentavalent antimonials for 30 days, following current WHO guidelines (*18*).

## Discussion

**Figure 6:**
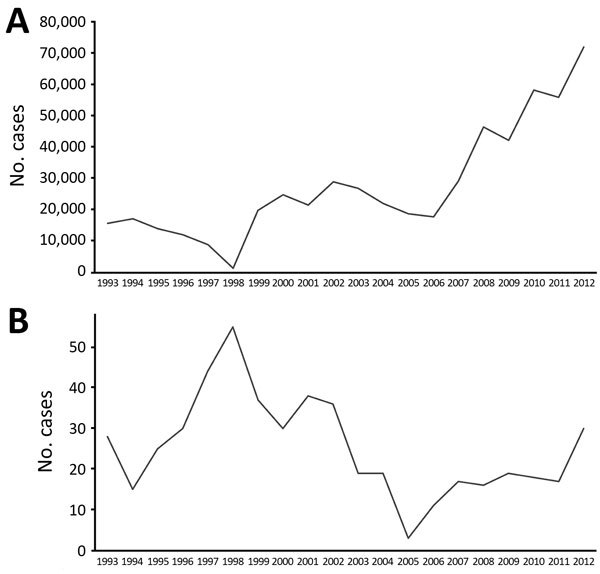
Cases of (A) cutaneous and (B) visceral leishmaniasis in northern Syria during 1993–2012, before the onset of the civil war (*1*,*17*).

Syria has been a hotspot of leishmaniasis transmission for centuries. After a continuous decrease of leishmanial transmission in the past century, which was driven at least in part by vector control activities of the national malaria elimination campaign, WHO country data show a gradual but steady increase in annual case incidence from ≈14,000 per year to an average of 27,825 reported CL cases/year before the onset of the civil war (*1**,**17*) (Figure 6).

The governmental health system in Syria disintegrated in most parts of the northern provinces after the onset of the civil war; at the same time, reports from neighboring regions indicated a surge in leishmaniasis cases in refugee populations from Syria. The MENTOR Initiative set out to collaborate with most local leishmaniasis treatment centers in the target provinces, becoming, in practice, the only external collaborating institution for leishmaniasis control in the target regions. It is assumed that the number of HCCs did not greatly affect the overall measure of incidence, because patients seeking ongoing treatment courses went to other operational centers within the program when needed. Based on this information and the comprehensive reporting system, the MENTOR Initiative was in a position to reliably assess and monitor the incidence of CL in northern Syria.

Thus, this survey provides epidemiologic data about the incidence of CL and VL in the war-affected regions of northern Syria since the onset of the civil war. Based on these data, a yearly incidence of 64,498 cases of CL was estimated, constituting a 2.6-fold increase in leishmaniasis transmission in the northern provinces over the ≈25,000 cases of CL reported by the national authorities before the civil war, in 2008 (Table 1). This increase is in line with several anecdotal reports about an observed surge of leishmaniasis cases in refugees from Syria (*2**–**4*).

These data indicate a noteworthy change in the epidemiology of leishmaniasis transmission on a regional level. The provinces of Aleppo, Hama, and Idlib were the traditional hotspots of CL endemicity, whereas the provinces of Al-Raqqa and Al-Hasakah reported relatively few cases before the civil war (*1*). The survey results we report demonstrate, in contrast to prewar data, that the most dramatic increase in leishmaniasis transmission occurred in regions of previously low transmission intensity, including a nearly 40-fold increase in incidence of cases in Al-Raqqa province. This change in the local epidemiology of leishmaniasis is most likely the result of a combination of factors, including large-scale population movement within the war-affected provinces and an increase in vector abundance as a result of an exponential increase in suitable sites for vector breeding caused by the barrel bombing of urban buildings, creating cracked walls and a buildup of domestic waste across cities and towns, conditions in which sand flies thrive and reproduce.

After the full implementation of this integrated leishmaniasis control program, estimated monthly case counts decreased progressively from March 2015 (n = 7,743) to February 2016 (n = 2,679). This change indicates a reduction of leishmaniasis transmission by >2-fold after the implementation of the control activities. Despite seasonal variations in incidence of leishmaniasis, most recent data indicate that control activities were able to reduce CL incidence to levels comparable to prewar conditions.

VL occurred sporadically during the study period; a total of 11 cases were reported by the participating HCCs. In contrast to the increases in CL, there was no notable increase in the reported incidence of VL in northern Syria at the time of this study, compared with prewar data. However, asymptomatic cases may have occurred but were unreported within the communities, and in some HCCs, related illnesses and deaths may have been misreported along with other unidentified causes of death.

Molecular analysis of leishmaniasis is recommended in high-resource settings to guide appropriate treatment. Previous data reported *L. tropica* as the main leishmanial species causing CL in northern Syria and *L. major* as the main species causing CL in southern Syria (*19**–**22*). This survey confirmed *L. tropica* as the predominant species in northern Syria, causing nearly 80% of cutaneous lesions. *L. major* was the second most prevalent species, accounting for 13% of all *Leishmania* species. *L. infantum* and *L. donovani*, which are considered classical pathogens for VL, were repeatedly confirmed in skin lesions of patients participating in this survey. In Syria, *L. infantum* has been reported previously as a causative agent of CL in humans (*21*); however, *L. donovani* had previously been isolated only from sand flies (*23*). Further, *L. donovani* has repeatedly been found in the neighboring countries Cyprus, Turkey, Iraq, and Israel (*24*–*28*). Recently, several cases of most likely autochthonous *L. donovani* infections have been reported from southeastern Anatolia in the direct vicinity of the border with Syria, and these were also cases of CL (*29*). In that study, *L. tropica* and *L. infantum* were described as the main causative agents of CL in Turkey, which fits well with our results for Syria. CL caused by *L. donovani* has also been documented from Cyprus (*26*). Moreover, numerous cases of CL caused by *L. donovani* have been documented from Sri Lanka (*30*). In summary, these molecular data demonstrate a surprising diversity of locally endemic *Leishmania* species causing cutaneous disease.

The challenging security situation led to several limitations in the conduct of the reported research activities. No accurate estimates of the actual population residing in the northern provinces were available because of the massive population movement within Syria, migration in neighboring countries, and casualties of the civil war. To address these limitations, we relied on approximations used by international institutions for this analysis. The safety situation made direct access to the participating HCCs in Syria impossible for external MENTOR personnel. Therefore, the control program and the epidemiologic assessments had to rely on collaborating local staff. Local healthcare workers traveled often under precarious safety situations to the border region with Turkey to receive training and supplies for the control activities within Syria. Because of these constraints, a more detailed investigation was not feasible, and the investigation had to be limited to aspects deemed of highest importance for the implementation and evaluation of the ongoing leishmaniasis control program.

It was within this challenging safety situation that the MENTOR Initiative developed and deployed this large leishmaniasis control program. Epidemiologic data indicate that the control program started in a period of rapidly rising leishmaniasis transmission in northern Syria during the civil war. A further increase in leishmaniasis transmission was avoided. However, political stability and a stable security situation are required, above all, to further stabilize and successfully control leishmaniasis in northern Syria.

Technical Appendix Details on universal *Leishmania* protocol and PCR assays.
