# Supplementary material for: Leishmaniasis in Northern Syria during Civil War
Source: Emerg Infect Dis. 2018 Nov;24(11):1973–81. doi: 10.3201/eid2411.172146 (PMC6199995; doi:10.3201/eid2411.172146)
Supplement: Technical Appendix — Details on universal Leishmania protocol and PCR assays. [file 17-2146-Techapp-s1.pdf]

Article DOI: https://doi.org/10.3201/eid2411.172146

Leishmaniasis in Northern Syria during the Civil War

Technical Appendix

Molecular Analysis of Skin Samples

Universal *Leishmania* PCR Protocol

The universal *Leishmania* PCR protocol uses the LITSR/L5.8S Primer Pair *(1)* followed by the protocol of Schönian et al. (*2*). This PCR amplifies a 300–350 bp fragment of the ribosomal repeat unit, including the first internal transcribed spacer (ITS1), length depending on species. We used 1 μL, 3 μL, and 6 μL of the extracted DNA for each sample. We used double-distilled DNA-free water (Sigma-Aldrich GmbH, Vienna, Austria) as a negative control. The amplicons were visualized in a 2% agarose gel by staining with GelRed (BioTrend, Cologne, Germany) and the amplified products were excised from the gel and purified with the Illustra Gel Band Purification Kit (GE Healthcare Bio-Sciences AB, Zipf, Austria). We sequenced all amplicons by direct sequencing using the BigDye sequencing kit and an automatic 310 ABI PRISM sequencer (PE Applied Biosystems, Darmstadt, Germany). Sequences were obtained from both strands and sequence data were processed with the GeneDoc sequence editor (www.nrbsc.org/gfx/genedoc/) to obtain consensus sequences. We compared individual sequences with previously published sequences from GenBank using a BLAST search.

Details on Other Assays

We also ran the K26-PCR assay (*3*), the cpbE/F-PCR assay (*4*,*5*), and the HSP70-PCR (*6*). We ran these PCRs with 3 μL and 6 μL if still enough material was available or only with 3 μL of extracted DNA and with a negative control (double-distilled molecular grade water). Unfortunately, no material was left from 3 of the samples identified as belonging to the *L. donovani/infantum* complex, and not enough material was left from several samples to run all PCRs and with all DNA concentrations. Amplicons were visualized by staining with ethidium bromide and electrophoresis in a 2% agarose gel and sequenced by direct DNA sequencing from the PCR product, as described in the preceding paragraph.

References

1. El Tai NO, Osman OF, El Fari M, Presber W, Schönian G. Genetic heterogeneity of ribosomal internal transcribed spacer in clinical samples of *Leishmania donovani* spotted on filter paper as revealed by single-strand conformation polymorphisms and sequencing. Trans R Soc Trop Med Hyg. 2000;94:575–9. PubMed http://dx.doi.org/10.1016/S0035-9203(00)90093-2

2. Schönian G, Nasereddin A, Dinse N, Schweynoch C, Schallig HD, Presber W, et al. PCR diagnosis and characterization of *Leishmania* in local and imported clinical samples. Diagn Microbiol Infect Dis. 2003;47:349–58. PubMed http://dx.doi.org/10.1016/S0732-8893(03)00093-2

3. Haralambous C, Antoniou M, Pratlong F, Dedet JP, Soteriadou K. Development of a molecular assay specific for the *Leishmania donovani* complex that discriminates *L. donovani*/*Leishmania infantum* zymodemes: a useful tool for typing MON-1. Diagn Microbiol Infect Dis. 2008;60:33–42. PubMed http://dx.doi.org/10.1016/j.diagmicrobio.2007.07.019

4. Hide M, Bañuls AL. Species-specific PCR assay for *L. infantum*/*L. donovani* discrimination. Acta Trop. 2006;100:241–5. PubMed http://dx.doi.org/10.1016/j.actatropica.2006.10.012

5. Zackay A, Nasereddin A, Takele Y, Tadesse D, Hailu W, Hurissa Z, et al. Polymorphism in the HASPB repeat region of east African *Leishmania donovani* strains. PLoS Negl Trop Dis. 2013;7:e2031. PubMed http://dx.doi.org/10.1371/journal.pntd.0002031

6. Van der Auwera G, Maes I, De Doncker S, Ravel C, Cnops L, Van Esbroeck M, et al. Heat-shock protein 70 gene sequencing for *Leishmania* species typing in European tropical infectious disease clinics. Euro Surveill. 2013;18:20543. PubMed http://dx.doi.org/10.2807/1560-7917.ES2013.18.30.20543
